# Cardiorespiratory effects of project energize: a whole-of region primary school nutrition and physical activity programme in New Zealand in 2011 and 2015

**DOI:** 10.1186/s13102-020-00200-0

**Published:** 2020-09-17

**Authors:** Carolyn Cairncross, Victor Obolonkin, Tara Coppinger, Elaine Rush

**Affiliations:** 1 School of Sport and Recreation, Auckland University of Technology, Auckland, New Zealand; 2 Department of Sport, Leisure & Childhood Studies, Cork Institute of Technology, Cork, Ireland

**Keywords:** Cardiorespiratory fitness, Nutrition, Physical activity, Intervention

## Abstract

**Background:**

Since 2004, Sport Waikato has delivered Project Energize, a through-school nutrition and physical activity program to primary schools in the Waikato. As part of the program’s continued assessment and quality control, the programme was evaluated in 2011 and 2015. This paper’s aim was to compare the cardiorespiratory fitness (time to run 550 m (T_550_)) levels of children participating in Project Energize in 2011 and 2015.

**Methods:**

In the 2011 evaluation of Project Energize, gender specific- T_550_-for-age Z scores (T_550_AZ) were derived from the T_550_ of 4832 Waikato children (2527 girls; 2305 boys; 36% Māori) aged between 6 and 12 years. In 2015, T_550_ was measured for 4798 (2361 girls; 2437 boys; 32% Māori) children, representative of age, gender and school socioeconomic status (SES). The T_550_AZ for every child in the 2015 study and 2011 evaluation were derived and differences in T_550_ between 2015 and 2011 by gender, SES and age were determined using independent t-tests. Multiple regression analysis predicted T550 Z score and run time, using year of measurement, gender, ethnicity, age and school SES.

**Results:**

With and without adjustment, children in 2015 ran 550 m faster than in 2011 (adjusted Z score 0.06, time 11 s). Specifically, girls ran at a similar speed in 2015 as 2011 but boys were faster than in 2011 (Z score comparison *P* < 0.001, mean difference 0.18 95%CI 0.12, 0.25). Regression analysis showed time taken to run 550 m was 11 s less in 2015 compared with 2011. Boys ran it 13 s faster than girls (Z score 0.07) and for each 1 year age increase, children were 8 s slower (Z score 0.006). For each 10% decrease in SES, children were 3 s slower (Z score 0.004) and Māori children were 5 s slower than Non-Māori children (Z score 0.15).

**Conclusions:**

The findings from this study support the continuation of the delivery of Project Energize in the Waikato region of New Zealand, as cardiorespiratory fitness scores in 2015, compared to 2011, were improved, particularly for lower SES schools and for Māori children. Ethnically diverse populations, schools with higher deprivation and girls, continue to warrant further attention to help achieve equity.

## Background

The prevalence of obesity, a form of malnutrition, is high (10%) in New Zealand children aged 2-14y [1]. Indigenous Māori children and those living in the most deprived areas are most at risk of obesity (16 and 20%, respectively) [1]. Since 2005, in response to the need for prevention strategies in childhood settings, [2] Sport Waikato, funded by the Waikato District Health Board, has delivered Project Energize (PE), a through-primary school nutrition and physical activity programme [3]. This is the first intervention of its kind to be delivered to primary school children in New Zealand. It began initially as a cluster randomised controlled trial in 124 schools in 2005 [4]. Briefly, an ‘Energizer’; a trained physical activity and nutrition change-agent works throughout the school year with lead teachers in 8 to 12 schools to achieve goals to provide healthier eating and quality physical activity opportunities. Example activities include the provision of professional development for teachers to teach fundamental movement skills [5], and the adaptation of the school environment to supply healthier foods and sports equipment upgrades. Since 2009, the intervention has reached all 252 primary schools in the Waikato region of New Zealand and its dose and specific components are tailored to each individual school [6]. The program’s fidelity is monitored by the funder with quarterly reports of activities of Energizers and schools submitted to the Waikato District Health Board.

Due to the requirement for healthcare evaluations to conform to the randomised controlled trial model [7], the translation of research findings is difficult as cost and time constraints are common given the complex settings of schools [8, 9]. Accountability both to children and funders (taxpayers) is required [10]. Frequently, changes in body mass index (BMI) alone have been used as the basis of such evaluations [11], however, an indicator of cardiorespiratory fitness, such as speed of running is also a marker of child health, [12] (particularly metabolic health), growth and development [13] and quality and quantity of physical activity [14, 15]. Given that evidence of effectiveness is poor in interventions incorporating physical activity, [16] an opportunity exists to utilise cardiorespiratory fitness data as a tool to effectively monitor and evaluate public-health interventions aimed at children.

A number of tests to measure physical fitness in school children have been developed, ranging from complex test batteries to simple run protocols [17]. The time taken to run 550 m (T_550_) has been validated as a measure of cardiorespiratory fitness [18]. In the 2011 evaluation of Project Energize (14), the BMI, waist-to-height ratio and percentage body fat of Waikato schoolchildren were positively associated with an increase in T550, and participating children ran faster than children from Canterbury in New Zealand [19]. As a result, it was proposed that T_550_ would be, in 2015, an alternative, pragmatic way to evaluate the effectiveness of Project Energize. This approach helped enable schools that were geographically remote, in areas of high deprivation and had ethnically diverse populations to participate in the evaluation [20].

Subsequently, the aim of this analysis was to compare cardiorespiratory fitness (T_550_) times of Waikato children in 2015 with children measured in the first large scale evaluation of the programme in 2011 [19]. These follow-up measures were undertaken in 2015 to assess maintenance of the programme, which had been delivered continuously in all schools for at least 6 years. As a result, it was hypothesised that the run times in 2015, relative to age and gender, would be maintained or faster than 2011.

## Methods

This retrospective study utilised two cross-sectional data sets of Waikato primary school children aged between 6 and 12 years. In 2011, 4832 children (2527 girls; 2305 boys; 36% Māori) from 193 (out of a total of 232) schools and in 2015, 4798 (2361 girls; 2437 boys; 32% Māori) children from 240 (out of 245) schools had time to run 550 m measured. The students in 2011 were not matched for students in 2015. However, this allowed for no child to be excluded; increasing participant numbers for analysis. For the 2011 measures, the Northern Y Regional Ethics Committee (NTY/10/04/41) provided ethical approval in 2010 and caregivers and children provided written and signed informed consent. In 2015, run times were measured in schools as part of a milestone report of effectiveness for the funder of PE (Waikato District Health Board). Anonymised run times and demographic information of gender, age in years and ethnicity, as recorded by schools as Māori or non-Māori, were provided for each child for the analysis. There were no inclusion/exclusion criteria for participation and all schools and children were invited to participate. The Auckland University of Technology ethics committee (AUTEC) provided written attestation that the analysis met ethical criteria. Socioeconomic status (SES) of each school was determined by the Ministry of Education on a scale of 1 to 10, with decile 1 schools located in the lowest, and decile 10 schools in the highest SES areas [21].

The standardised protocol for T_550_ was followed [22] . In brief, after a standardised warm up, time taken for children to run five times around an outdoor, grassy, 110 m oval course (26.5 m by 42.5 m) was recorded (±1 s). A maximum of 4–5 children ran together in groups according to their age (school year) and self-identified speed. Previous investigators have reported moderate to strong reliability and validity of the 550 m (600 yards) run [17, 23] and in our hands (unpublished) we have shown that the different day intraindividual variation is ±6 s ~ 2.5%.

Data collected in 2011 and 2015 were used for this study as these were the only 2 years where run scores were measured and evaluated in a co-ordinated fashion. As boys and girls grow and develop at different rates at different ages, “measure-for-age” charts are needed to be able to compare measurements of children with a reference population. From the previously developed 2011 run times [24], gender-specific T_550_ charts were published allowing the determination of T_550_-for-age Z scores (T_550_AZ) for a child or group of children. For the 2015 children, as birthdate was not provided, median-age-within-year was used e.g. all 6 year olds were recorded as 6.5 years. Differences in T_550_ AZ between 2015 and 2011 were determined using independent t-tests. Mean T_550_AZ and 95% confidence intervals of the mean differences are described and T_550_AZ scores compared systematically by year of measurement, gender, ethnicity and school decile (Table 2).

All continuous data was examined for outliers and tested for normality. Z scores for age and gender adjusted run times are reported as mean and standard deviation. Differences in Z scores T_550_ by year, gender, ethnicity and decile and year of measurement were determined by unpaired t tests. Magnitude of difference was determined from the 95% CI of the mean difference. Multiple linear regression analyses to determine associations of age, gender, ethnicity, decile and year of measurement with both Z scores and time in seconds. All comparisons were two-sided with statistical significance was set at *P* < 0.05. Statistical analysis was undertaken using Statistical Package for the Social Sciences (SPSS, v22, IBM).

## Results

In 2015, children ran 550 m faster than in 2011 (Z score 0.11, Table 1). Specifically, girls ran at a similar speed in 2015 as 2011 but boys were faster than in 2011 (Z score comparison *P* < 0.001, mean difference 0.18 95%CI 0.12, 0.25). This unadjusted difference was also seen in all children aged 6 (0.19; 0.01,0.37, *p* = 0.037), 8 (0.18; 0.06,0.30, *p* = 0.0005), and 9 (0.29; 0.28,0.40, *p* < 0.001) years.

In the regression analyses adjusting for age, gender, decile, ethnicity and year of measurement, time taken to run 550 m was 11 s (Z score 0.06) less in 2015 compared with 2011 (Tables 2 and 3). Boys ran 550 13 s faster than girls (Z score 0.07) and for each 1 year age increase, children were 8 s faster (Z score 0.006; Table 3). For each 10% decrease in SES, children were 3 s slower (Z score 0.004) and Māori children were 5 s slower than Non-Māori children (Z score 0.15).

**Table 1:** Z score differences in time to run 550 m (T_550_) of Waikato primary school children in 2015 and 2011

Characteristic	2015 (*n* = 4798)		2011 (*n* = 4832)		Difference Z score (2011 minus 2015)	
	n	T_550_AZ mean (SD)	n	T_550_AZ mean (SD)	P	Difference (95%CI)^a^
All	4798	−0.11 (1.236)		0.00 (1.00)	**< 0.001**	**0.11 (−0.15, − 0.06)**
Gender						
Boys	2437	− 0.18 (1.26)	2305	0.00 (1.00)	**< 0.001**	**0.18 (0.12, 0.25)**
Girls	2361	−0.04 (1.21)	2527	0.00 (1.00)	0.14	0.04 (−0.02, 0.10)
Age						
6 years	432	−0.10 (1.48)	358	0.09 (0.98)	**0.037**	**0.19 (0.01, 0.37)**
7 years	1118	−0.09 (1.29)	1683	−0.05 (1.00)	0.36	0.04 (−0.05, 0.13)
8 years	978	−0.11 (1.24)	539	0.07 (1.04)	**0.005**	**0.18 (0.06, 0.30)**
9 years	973	−0.21 (1.14)	582	0.08 (1.02)	**< 0.001**	**0.29 (0.18, 0.40)**
10 years	1297	−0.05 (1.16)	1670	−0.03 (0.98)	0.13	0.02 (−0.06, 0.10)
Ethnicity						
All Māori	1516	0.16 (1.25)	1905	0.24 (0.98)	**0.036**	**0.08 (0.01, 0.15)**
All Non-Māori	3282	−0.24 (1.21)	2927	−0.16 (0.98)	**0.005**	**0.08 (0.02,0.14)**
Māori boys	801	0.13 (1.25)	908	0.27 (0.96)	**0.001**	**0.14 (0.03, 0.25)**
Māori girls	715	0.20 (1.25)	997	0.21 (1.02)	0.85	0.01 (− 0.98, 0.12)
Non-Māori boys	1636	−0.32 (1.25)	1397	− 0.18 (0.99)	**< 0.001**	**0.14 (0.06, 0.22)**
Non-Māori girls	1646	−0.14 (1.18)	1530	−0.14 (0.96)	1.00	0.00 (−0.07, 0.07)
SES						
Decile 1^b^	439	0.71 (1.12)	450	0.47 (0.93)	**0.002**	**−0.24 (− 0.38, − 0.11)**
Decile 2	435	0.59 (1.15)	615	0.31 (0.99)	**0.006**	**−0.28 (− 0.41, − 0.15)**
Decile 3	629	0.06 (1.17)	654	0.16 (0.97)	0.095	0.10 (−0.02, 0.22)
Decile 4	306	−0.25 (1.12)	745	0.05 (0.96)	**0.002**	**0.30 (0.17, 0.44)**
Decile 5	716	−0.23 (1.27)	397	−0.10 (0.96)	0.076	0.13 (−0.01, 0.27)
Decile 6	501	−0.29 (1.15)	479	−0.04 (0.94)	**< 0.001**	**0.25 (0.12, 0.38)**
Decile 7	475	−0.35 (1.17)	346	−0.16 (0.94)	**0.013**	**0.19 (0.04, 0.34)**
Decile 8	611	−0.45 (1.21)	404	−0.32 (0.93)	0.067	0.13 (−0.01. 0.27)
Decile 9	352	−0.43 (1.13)	319	−0.25 (0.99)	**0.029**	**0.18 (0.02, 0.34)**
Decile 10	334	−0.48 (1.11)	423	−0.53 (0.97)	0.51	−0.05 (− 0.20, 0.09)

T_550_ time to run 550 m, T_550_AZ gender specific for-age z-scores

SD standard deviation

^a^Independent t-test, a positive Z score difference indicates a decrease in time to run in 2015 compared with 2011

^b^School decile as determined by the Ministry of Education, scale 1–10: 1 located in lowest, and 10 in highest, socioeconomic area^21^

**Table 2:** Multiple regression analysis for time to complete in seconds T550 using year of measurement, gender, ethnicity, age and decile

Characteristic	Unstandardized Coefficients			Standardized Coefficients	
	Beta sec	Std.Error	Difference (95%CI)	Beta	P
Constant	264.1	2.3	260.1, 268.1		< 0.001
Age	−7.959	0.2	−8.4, − 7.5	−0.321	**< 0.001**
Gender	−13.0	0.6	−14.2, −11.8	−0.189	**< 0.001**
Decile	−2.9	0.1	−3.2, − 2.7	−0.234	**< 0.001**
Ethnicity	−4.4	0.7	−5.8, −3.0	−0.062	**< 0.001**
Year	11.3	0.6	10.1, 12.6	0.165	**< 0.001**

Gender Girl 0, Boy 1; Decile linear 1 to 10, Ethnicity 0 Māori, 1 Non-Māori, Year 2011 = 0, 2015 = 1

**Table 3:** Multiple regression analysis for prediction of T550 z score using year of measurement, gender, ethnicity, age and decile

Characteristic	Unstandardized Coefficients			Standardized Coefficients	
	B	Std.Error	(95%CI)	Beta	P
Constant	0.682	0.072	0.541, 0.824		< 0.001
Age, y	−0.006	−0.008	−0.021, − 0.010	−0.007	0.482
Gender	−0.075	−0.022	− 0.118, − 0.032	−0.033	**< 0.005**
Decile	−0.102	−0.004	− 0.110, − 0.093	−0.248	**< 0.001**
Ethnicity	−0.151	−0.025	− 0.201, − 0.102	−0.064	**< 0.001**
Year	0.058	0.022	−0.101, − 0.015	−0.026	**< 0.01**

Gender Girl 0, Boy 1; Decile linear 1 to 10, Ethnicity 0 Māori, 1 Non-Māori, Year 2011 = 0, 2015 = 1

## Discussion

The aim of this 2018 analysis was to compare cardiorespiratory fitness (T_550)_ levels between 2015 and 2011 of Waikato children participating in Project Energize. Overall, the run scores were faster in 2015 compared to 2011. This is an encouraging statistic and suggests that the PE intervention has maintained the increase in quality physical activity in schools reported previously [5]. A recent meta-analysis of the effect of school-based physical activity interventions aimed at increasing cardiorespiratory fitness found moderate quality evidence supporting their effectiveness [25]. However, this increase was only found in girls, which is in contrast to the findings of this current study. Given that Energizers tailor physical activities to the needs of individual schools may help explain these differences.

In the Waikato region between the periods 2011–2014 and 2014–2017, overweight and obesity prevalence decreased from 36.7%(95%CI, 32.7, 40.9) to 29.2%(25.5, 33.2) [26]. In the same period, there was a small increase for the whole of New Zealand. This suggests that the decrease in body size found in this study may be part of the explanation of the faster run times, supported by the delivery of PE in all Waikato primary schools. Previous research has reported that children whose body fat percentage increases during a physical activity intervention are more likely to also have a decrease in cardiorespiratory fitness [25]. Yet, it can’t be ignored that Māori children, schools with higher deprivation and girls, continue to warrant attention. It has been hypothesized that future intervention programs may need to be more aggressive when it comes to positively impacting cardiorespiratory fitness via school-based interventions [25]. Targeting girls [27] and schools that are ethnically diverse [28] and have lower socio-economic status [29] may also need to consider alternative approaches.

In addition to the large number of schools who participated, there are several strengths of this study. Firstly, the inclusion of a valid physical fitness measure widens the outcome measures for programme evaluation. Secondly, the anonymous collection of data as part of each school’s curriculum is part of the quality control of delivery and reduces participant bias. From a cost perspective, this parsimonious and pragmatic measurement requires minimal equipment and training, and can be repeated with large numbers of children; including those that are geographically remote, in areas of high deprivation and have ethnically diverse populations. Limitations of this study include its cross-sectional design and the inability to identify the contribution of specific components of the PE program to the cardiorespiratory fitness scores recorded. Although our analysis did not take into account the size of the school, clustering effects or years of attendance of child at the school, the large number of children tested and inclusion of more than 80% of the schools in the region in 2011 and 2015 meant that there was good representation of the region.

Although program adherence data was not collected between 2011 and 2015 for each individual school, adherence was reported to the funder by all Energizers in the form of quarterly reports for the region. This report described overall, the number of interactions and activities undertaken.

## Conclusion

The findings from this study support the continuation of the delivery of Project Energize in the Waikato region of New Zealand, as cardiorespiratory fitness scores in 2015, compared to 2011, were improved, particularly for lower SES schools and for Māori children. Ethnically diverse populations, schools with higher deprivation and girls, continue to warrant further attention to help achieve equity.

## Data Availability

The dataset supporting the conclusions of this article are available by contacting Professor Elaine Rush directly.

## Abbreviations

T_550_AZ: T550-for-age Z scores
SES: School socioeconomic status
PE: Project Energize
BMI: Body mass index
AUTEC: Auckland University of Technology ethics committee
